# Supplementary material for: ADARs act as potent regulators of circular transcriptome in cancer
Source: Nat Commun. 2022 Mar 21;13:1508. doi: 10.1038/s41467-022-29138-2 (PMC8938519; doi:10.1038/s41467-022-29138-2)
Supplement: Supplementary file 1 — Supplementary Information [file 41467_2022_29138_MOESM1_ESM.pdf]

Supplementary Information for

ADARs act as potent regulators of circular transcriptome in cancer

Haoqing Shen, Omer An, Xi Ren, Yangyang Song, Sze Jing Tang, Xin-Yu Ke, Jian Han, Daryl Jin Tai Tay, Vanessa Hui En Ng, Fernando Bellido Molias, Priyankaa Pitcheshwar, Ka Wai Leong, Ker-Kan Tan, Henry Yang & Leilei Chen*

*Correspondence to: Leilei Chen, polly_chen@nus.edu.sg; Tel: +65 6516 8435; Fax: +65 6516 1873.

This PDF file includes:

Supplementary Figures 1-4

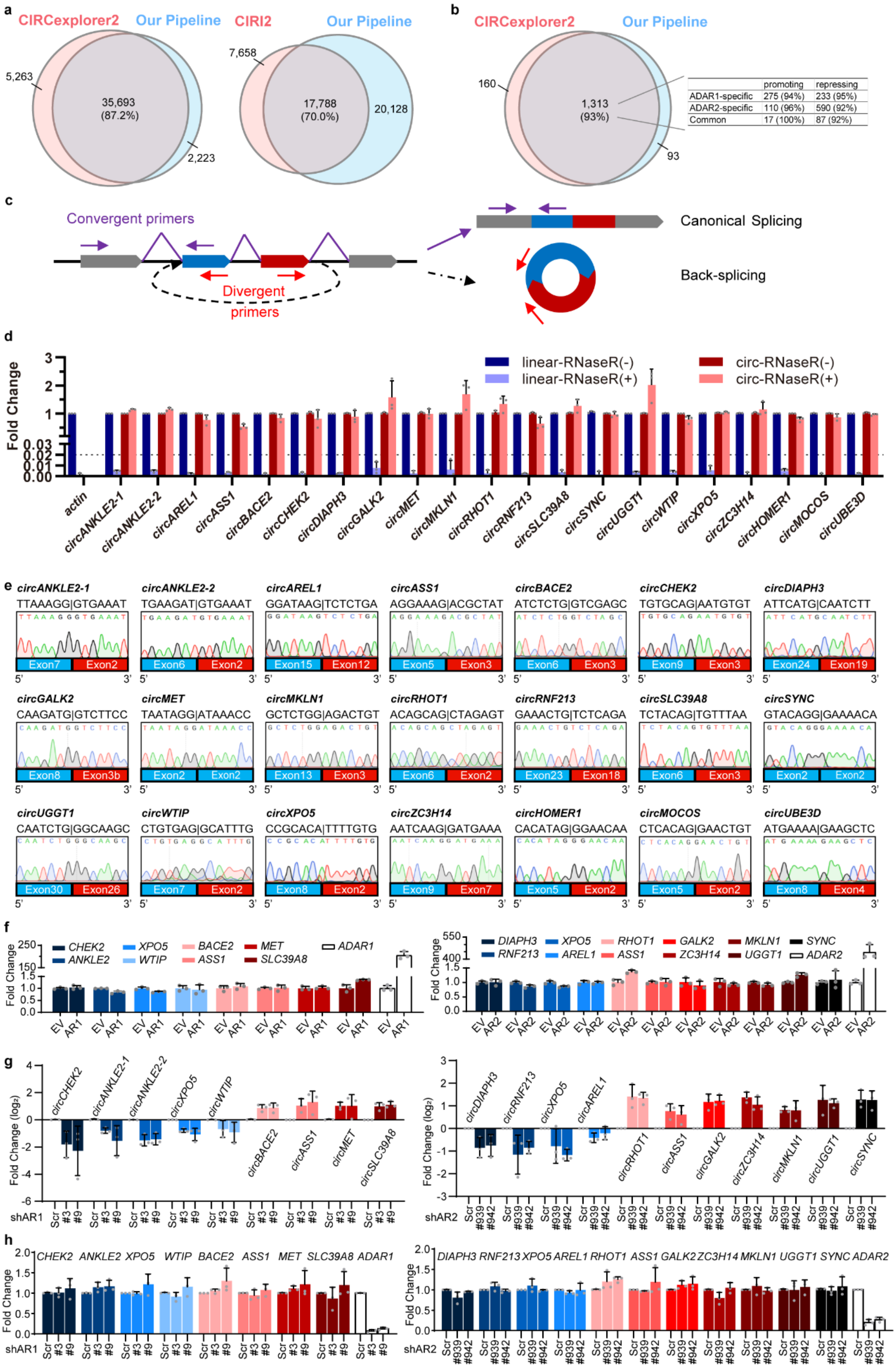
a
CIRCexplorer2
Our Pipeline
5,263
35,693
(87.2%)
2,223
CIRI2
Our Pipeline
7,658
17,788
(70.0%)
20,128
b
CIRCexplorer2
Our Pipeline
160
1,313
(93%)
93
promoting repressing
ADAR1-specific 275 (94%) 233 (95%)
ADAR2-specific 110 (96%) 590 (92%)
Common 17 (100%) 87 (92%)
c
Convergent primers
Divergent primers
Canonical Splicing
Back-splicing
d
Fold Change
linear-RNaseR(-)
linear-RNaseR(+)
circ-RNaseR(-)
circ-RNaseR(+)
actin
circANKLE2-1
circANKLE2-2
circAREL1
circASS1
circBACE2
circCHEK2
circDIAPH3
circGALK2
circMET
circMKLN1
circRHOT1
circRNF213
circSLC39A8
circSYNC
circUGGT1
circWTIP
circXPO5
circZC3H14
circHOMER1
circMOCOS
circUBE3D
e
circANKLE2-1 TTAAAGG|GTGAAAT Exon7 Exon2
circANKLE2-2 TGAAGAT|GTGAAAT Exon6 Exon2
circAREL1 GGATAAG|TCTCTGA Exon15 Exon12
circASS1 AGGAAAG|ACGCTAT Exon5 Exon3
circBACE2 ATCTCTG|GTCGAGC Exon6 Exon3
circCHEK2 TGTGCAG|AATGTGT Exon9 Exon3
circDIAPH3 ATTCATG|CAATCTT Exon24 Exon19
circGALK2 CAAGATG|GTCTTCC Exon8 Exon3b
circMET TAATAGG|ATAAACC Exon2 Exon2
circMKLN1 GCTCTGG|AGACTGT Exon13 Exon3
circRHOT1 ACAGCAG|CTAGAGT Exon6 Exon2
circRNF213 GAAACTG|TCTCAGA Exon23 Exon18
circSLC39A8 TCTACAG|TGTTTAA Exon6 Exon3
circSYNC GTACAGG|GAAAACA Exon2 Exon2
circUGGT1 CAATCTG|GGCAAGC Exon30 Exon26
circWTIP CTGTGAG|GCATTTG Exon7 Exon2
circXPO5 CCGCACA|TTTTGTG Exon8 Exon2
circZC3H14 AATCAAG|GATGAAA Exon9 Exon7
circHOMER1 CACATAG|GGAACAA Exon5 Exon2
circMOCOS CTCACAG|GAACTGT Exon5 Exon2
circUBE3D ATGAAAA|GAAGCTC Exon8 Exon4
f
Fold Change
CHEK2 ANKLE2 XPO5 WTIP BACE2 ASS1 MET SLC39A8 ADAR1
EV AR1
DIAPH3 RNF213 XPO5 AREL1 RHOT1 ASS1 GALK2 ZC3H14 MKLN1 UGGT1 SYNC ADAR2
EV AR2
g
Fold Change (log2)
circCHEK2 circANKLE2-1 circANKLE2-2 circXPO5 circWTIP circBACE2 circASS1 circMET circSLC39A8
shAR1 Scr #3 #9
circDIAPH3 circRNF213 circXPO5 circAREL1 circRHOT1 circASS1 circGALK2 circZC3H14 circMKLN1 circUGGT1 circSYNC
shAR2 Scr #939 #942
h
Fold Change
CHEK2 ANKLE2 XPO5 WTIP BACE2 ASS1 MET SLC39A8 ADAR1
shAR1 Scr #3 #9
DIAPH3 RNF213 XPO5 AREL1 RHOT1 ASS1 GALK2 ZC3H14 MKLN1 UGGT1 SYNC ADAR2
shAR2 Scr #939 #942

Supplementary Fig. 1. ADAR1 and ADAR2 regulate circRNA biogenesis in both directions without affecting their host gene expression.

a, Venn diagram showing the overlapping circRNAs detected by our pipeline and CIRCexplore2 (left panel) or CIRI2 (right panel). The numbers showed in brackets indicate the overlapping percentage in circRNAs detected by CIRCexplorer2 or CIRI2.

b, Venn diagram and table showing the overlapping ARcircs detected by our pipeline and CIRCexplore2. The numbers showed in brackets indicate the overlapping percentage in ARcircs detected by our pipeline.

c, Schematic diagram illustrating primer design strategy for detecting circRNAs and linear RNAs.

d, qRT-PCR validations of the indicated circRNAs and their host linear mRNA transcripts, with or without RNaseR digestion.

e, Sequence chromatograms showing the sequences at back-splicing junctions (BSJ) of all 21 circRNA targets selected for validation.

f, qPCR analysis showing the fold change in expression level of each indicated host gene transcript, upon overexpression of ADAR1 (AR1, left panel) or ADAR2 (AR2, right panel) *versus* the empty vector (EV) control. Data are presented as the mean ± S.D. of technical triplicates from a representative experiment of 2 independent experiments.

g, h, qPCR analysis showing fold change in expression level of each indicated circRNA (**g**) and their corresponding host gene transcript (**h**), upon knockdown of ADAR1 (shAR1#3 and #9) or ADAR2 (shAR2#939 and #942), when compared to the scramble control (shScr).

(d,g,h) Each dot represents the mean value of technical triplicates from an independent experiment. Data are presented as the mean ± S.D. of 3 biological replicates.

Source data are provided in Source Data file.

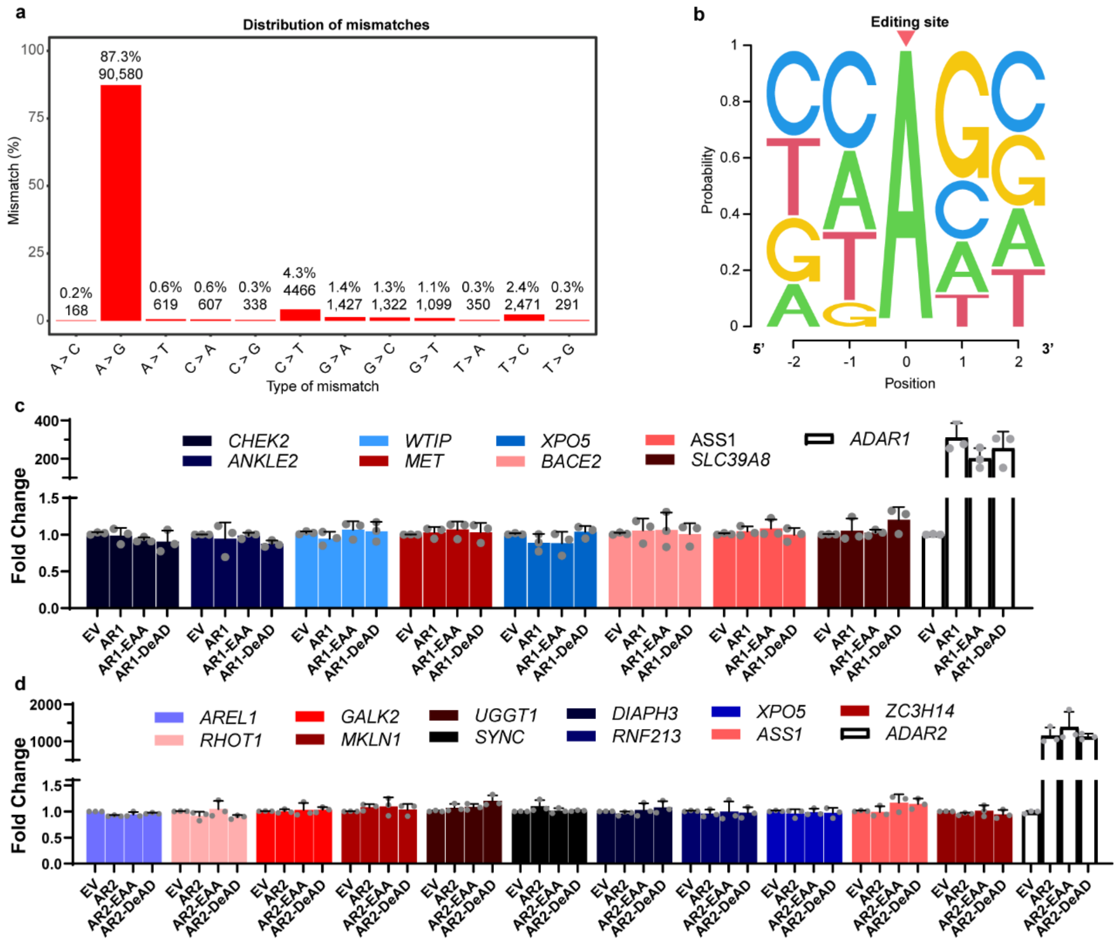

Supplementary Fig. 2. Overexpression of mutant ADAR1/2 has no obvious effect on the host gene expression in EC109 cells.

a. Distribution of 12 types of mismatches from RNA sequencing data.

b. Sequence preference of 2 neighbouring nucleotides surrounding A-to-I editing sites.

c,d, qRT-PCR analysis of fold change in expression level of each indicated host linear mRNA, upon overexpression of the WT or mutant form of ADAR1 (**c**) or ADAR2 (**d**) *versus* the EV control. Each dot represents the mean value of technical triplicates. Data are presented as the mean ± S.D. of 3 biological replicates.

Source data are provided in Source Data file.

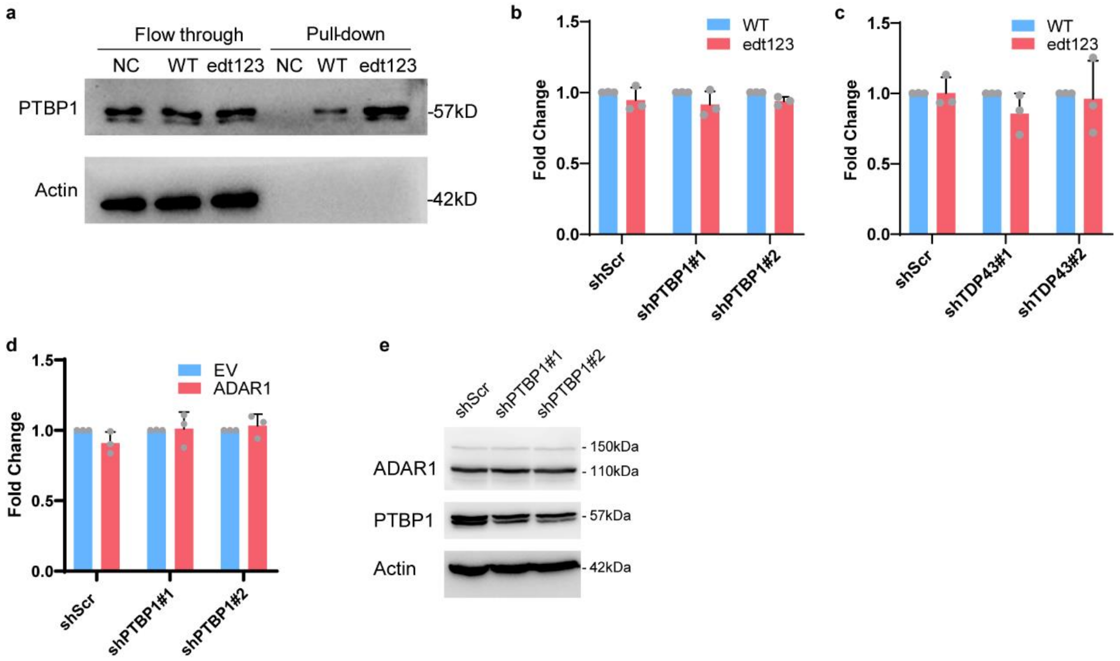

Supplementary Fig. 3. PTBP1 binds to edited RCM to regulate *circCHEK2* without affecting linear *CHEK2* and ADAR1 expression.

a, WB analysis of RNA pulldown and flow through samples showing the binding affinity of PTBP1 to the indicated RNA probes. NC, the RNA probe with all 12 PTBP1 binding motifs mutated. WT, the wildtype probe. Edt123, the RNA probe with A-to-G mutation at each of three editing sites. Representative result of $n = 2$.

b,c,d, qRT-PCR analysis of fold change in expression level of minigene-derived linear *CHEK2* transcript (**b, c**) or endogenous linear *CHEK2* transcript (**d**) in the indicated samples. Each dot represents the mean of 3 independent experiments. Data are presented as the mean ± S.D. of 3 biological replicates.

e, WB analysis of the indicated proteins in EC109 cells upon PTBP1 knockdown. Actin was used as a loading control. Representative result of $n = 2$.

Source data are provided in Source Data file.

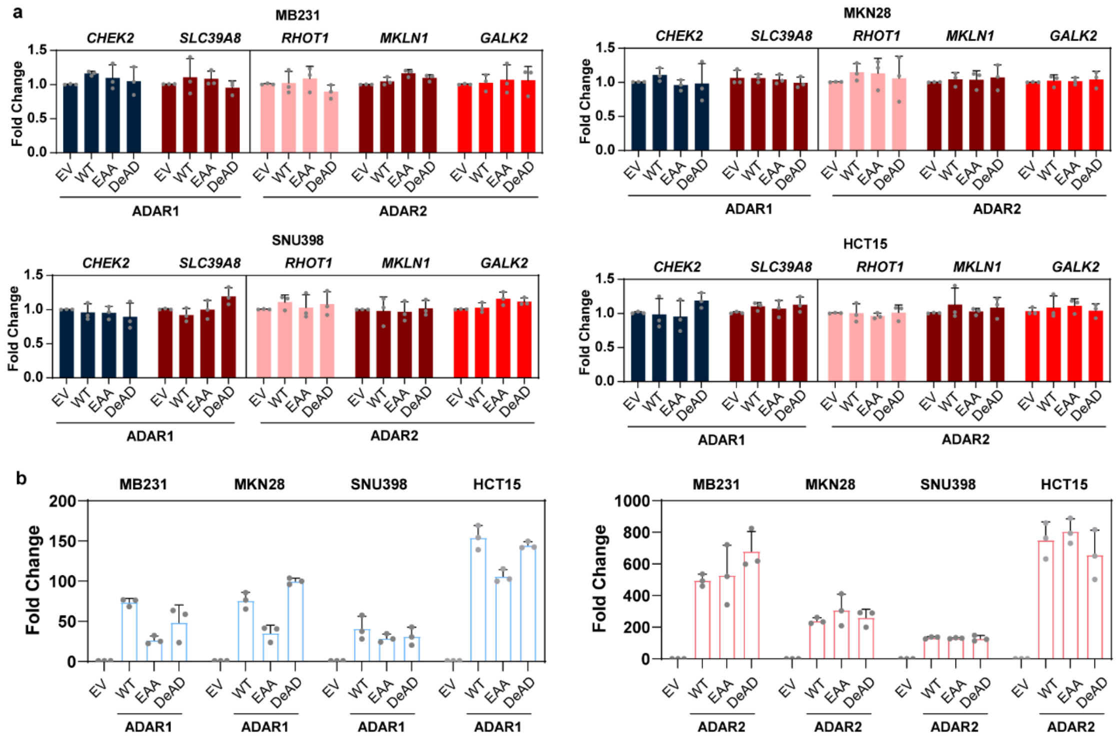

Supplementary Fig. 4. ADAR-mediated circRNA regulation exists in multiple cancer cell lines.

a, qRT-PCR analysis of fold change in expression level of each indicated host linear mRNA, upon overexpression of the WT or mutant ADAR1 or ADAR2 *versus* the EV control in MB231, MKN28, SNU398, and HCT15 cells.

b, qRT-PCR analysis of fold change in expression level of WT or mutant ADAR1 or ADAR2 *versus* the EV control in MB231, MKN28, SNU398, and HCT15 cells.

(a,b), Each dot represents the mean of 3 independent experiments. Data are presented as the mean ± S.D. of biological triplicates.

Source data are provided in Source Data file.
