# Supplementary material for: ADARs act as potent regulators of circular transcriptome in cancer
Source: Nat Commun. 2022 Mar 21;13:1508. doi: 10.1038/s41467-022-29138-2 (PMC8938519; doi:10.1038/s41467-022-29138-2)
Supplement: Supplementary file 3 — Description of Additional Supplementary Files [file 41467_2022_29138_MOESM3_ESM.pdf]

Description of Additional Supplementary Files

File Name: Supplementary Data 1
Description: List of ADAR1/2-regulated circRNAs identified from circRNA-seq using our in-house pipeline and CIRCexplorer2. Related to Figure 1 and Supplementary Figure 1.

File Name: Supplementary Data 2
Description: Predicted RCMs of ADAR1/2-regulated circRNAs. Related to Figure 2.

File Name: Supplementary Data 3
Description: List of identified editing-dependent and -independent regulated ARcircs.

File Name: Supplementary Data 4
Description: List of RBPs and the number of circRNAs with altered RBP binding sites due to editing. Related to Figure 5.

File Name: Supplementary Data 5
Description: List of primers, shRNAs, guide RNAs, sequence input for secondary structure prediction and RNA probes.

File Name: Supplementary Software 1
Description: The codes and software used for bioinformatics analyses in this study.
