# Supplementary material for: ADARs act as potent regulators of circular transcriptome in cancer
Source: Nat Commun. 2022 Mar 21;13:1508. doi: 10.1038/s41467-022-29138-2 (PMC8938519; doi:10.1038/s41467-022-29138-2)
Supplement: Supplementary file 10 — Reporting Summary [file 41467_2022_29138_MOESM10_ESM.pdf]

nature portfolio

Corresponding author(s): Leilei Chen

Last updated by author(s): Jan 26, 2022

Reporting Summary

Nature Portfolio wishes to improve the reproducibility of the work that we publish. This form provides structure for consistency and transparency in reporting. For further information on Nature Portfolio policies, see our Editorial Policies and the Editorial Policy Checklist.

Statistics

For all statistical analyses, confirm that the following items are present in the figure legend, table legend, main text, or Methods section.

n/a	Confirmed	
☐	☒	The exact sample size (*n*) for each experimental group/condition, given as a discrete number and unit of measurement
☐	☒	A statement on whether measurements were taken from distinct samples or whether the same sample was measured repeatedly
☐	☒	The statistical test(s) used AND whether they are one- or two-sided<br>*Only common tests should be described solely by name; describe more complex techniques in the Methods section.*
☒	☐	A description of all covariates tested
☒	☐	A description of any assumptions or corrections, such as tests of normality and adjustment for multiple comparisons
☐	☒	A full description of the statistical parameters including central tendency (e.g. means) or other basic estimates (e.g. regression coefficient) AND variation (e.g. standard deviation) or associated estimates of uncertainty (e.g. confidence intervals)
☐	☒	For null hypothesis testing, the test statistic (e.g. *F*, *t*, *r*) with confidence intervals, effect sizes, degrees of freedom and *P* value noted<br>*Give P values as exact values whenever suitable.*
☒	☐	For Bayesian analysis, information on the choice of priors and Markov chain Monte Carlo settings
☒	☐	For hierarchical and complex designs, identification of the appropriate level for tests and full reporting of outcomes
☒	☐	Estimates of effect sizes (e.g. Cohen's *d*, Pearson's *r*), indicating how they were calculated

Our web collection on statistics for biologists contains articles on many of the points above.

Software and code

Policy information about availability of computer code

Data collection	For identification of ADAR-regulated circRNAs (ARcircs), the expression level of ADAR1 or ADAR2 was modulated (forced overexpression or silencing) using a lenti-viral system in EC109 cells. RNA extraction was performed using RNeasy Mini kit (Qiagen) and subjected to rRNA depletion and RNase R treatment to digest all the linear RNAs. Samples were sequenced on an Illumina HiSeq4000 instrument with 100-bp paired end reads.<br>For identification of editing-dependent ARcircs, we overexpressed the catalytic mutants of ADAR1 or ADAR2 (ADAR1-DeAD or ADAR2-DeAD) or the empty vector (EV) control in EC109 cells and performed circRNA-Seq using Illumina NovaSeq6000 instrument with 100-bp paired end reads.
Data analysis	Raw reads were mapped to the reference human genome (hg19) by STAR (v2.5.2a) for in-house circRNA identification pipeline. CIRI2 (v2.0.6) and CIRCexplorer2 (v2.3.0) was used as benchmark methods. CSI NGS Portal (https://csibioinfo.nus.edu.sg/csingsportal) was used to identify RNA editing events from total RNA-Seq data. The sequence logo was plotted by using the "seqLogo" package (v1.56.0). A BLAST alignment was performed for each intron pair flanking circRNA junctions to identify all the potential candidates. RNAfold (v2.4.18) was used for RNA secondary structure prediction. RBPmap (version 1.2) was used for RBP motif analysis. ImageJ (1.51J8) was used to measure band density of blots. OpenCFU (v3.9.0) was used for foci number calculation. Graphpad Prism 8.2.0 was used for statistics analysis and graph preparation.

For manuscripts utilizing custom algorithms or software that are central to the research but not yet described in published literature, software must be made available to editors and reviewers. We strongly encourage code deposition in a community repository (e.g. GitHub). See the Nature Portfolio guidelines for submitting code & software for further information.

Data

Policy information about availability of data

All manuscripts must include a data availability statement. This statement should provide the following information, where applicable:

- Accession codes, unique identifiers, or web links for publicly available datasets
- A description of any restrictions on data availability
- For clinical datasets or third party data, please ensure that the statement adheres to our policy

The circRNA-Seq data generated in this study have been deposited in the Gene Expression Omnibus (GEO) under accession code GSE164681 [https://www.ncbi.nlm.nih.gov/geo/query/acc.cgi?acc=GSE164681]. The EC109 RNA-Seq data has been published previously and are also available at GEO under accession [https://www.ncbi.nlm.nih.gov/geo/query/acc.cgi?acc=GSE131658]. Human genome reference hg19 was obtained from GENCODE [https://www.gencodegenes.org/]. A-to-I editing sites from RADAR database were obtained from http://RNAedit.com. Information of SNPs was obtained from 1000 Genomes Project [https://www.internationalgenome.org/], NHLBI GO Exome Sequencing Project [http://evs.gs.washington.edu/EVS/], and dbSNP v138 [https://www.ncbi.nlm.nih.gov/SNP/]. Source data are provided as a Source Data file.

Field-specific reporting

Please select the one below that is the best fit for your research. If you are not sure, read the appropriate sections before making your selection.

☒ Life sciences ☐ Behavioural & social sciences ☐ Ecological, evolutionary & environmental sciences

For a reference copy of the document with all sections, see nature.com/documents/nr-reporting-summary-flat.pdf

Life sciences study design

All studies must disclose on these points even when the disclosure is negative.

Sample size	Sample sizes for each experiment are provided in figure legends. For in vivo tumorigenicity assay, n=5 (SNU398) or n=6 (EC109) mice were used. The sample sizes were determined based on common practice in the field.
Data exclusions	No data were excluded from analysis
Replication	Replicate experiments were successful. The number of replication for each experiments were indicated in figure legends.
Randomization	Cells were randomly allocated into each group in relevant experiments.
Blinding	The investigators were not blinded as proper controls were already included during experiment design.

Reporting for specific materials, systems and methods

We require information from authors about some types of materials, experimental systems and methods used in many studies. Here, indicate whether each material, system or method listed is relevant to your study. If you are not sure if a list item applies to your research, read the appropriate section before selecting a response.

Materials & experimental systems

n/a	Involved in the study	
☐	☒	Antibodies
☐	☒	Eukaryotic cell lines
☒	☐	Palaeontology and archaeology
☐	☒	Animals and other organisms
☐	☒	Human research participants
☒	☐	Clinical data
☒	☐	Dual use research of concern

Methods

n/a	Involved in the study	
☒	☐	ChIP-seq
☒	☐	Flow cytometry
☒	☐	MRI-based neuroimaging

Antibodies

Antibodies used	Anti-PTBP1, Abcam Cat# ab133734, (1:1,000 dilution)<br>Anti-TDP43, Proteintech Cat# 10782-2-AP, (1:1,000 dilution)<br>Anti-ADAR1, Abcam, Cat# ab88574, (1:1,000 dilution)<br>Anti-FLAG-HRP, Sigma, Cat# A8592, (1:1,000 dilution)<br>Anti-beta actin-HRP, Santa Cruz Biotechnology Cat# sc-47778HRP, (1:10,000 dilution)

	Anti-mouse IgG, HRP-linked, Cell Signaling Technology Cat# 7076; RRID:AB_330924, (1:10,000 dilution)<br>Anti-rabbit IgG, HRP-linked, Cell Signaling Technology Cat#7074; RRID:AB_2099233, (1:10,000 dilution)
Validation	All primary antibodies were validated for western-blot in human cells. Validation details can be found on manufacturer's website. Anti-PTBP1, Abcam Cat# ab133734, was validated for RIP experiment in published paper (Cheng et al., J Cell Mol Med. 2020 May; 24(9): 5274–5289.)

Eukaryotic cell lines

Policy information about cell lines

Cell line source(s)	Human EC109 cells, Chinese National Infrastructure of Cell Line Resource, Cat# 1101HUM-PUMC000246<br>Human SNU398 cells, American Type Culture Collection (ATCC), Cat# CRL-2233<br>Human MKN28 cells, Japanese Collection of Research Bioresources Cell Bank, Cat# JCRB0253<br>Human MB231 cells, ATCC, Cat# HTB-26<br>Human HCT15 cells, ATCC, Cat# CCL-225
Authentication	None of the cell lines used were authenticated.
Mycoplasma contamination	Mycoplasma contamination was not found in these cell lines.
Commonly misidentified lines<br>(See ICLAC register)	MKN28, although commonly misidentified, the contaminating cell line MKN74 is also a gastric carcinoma cell line, which is suitable in our study.

Animals and other organisms

Policy information about studies involving animals; ARRIVE guidelines recommended for reporting animal research

Laboratory animals	4–6 weeks old NOD scid gamma (NSG) mice (for EC109, 6 female; for SNU398, 4 female +1 male) were used.
Wild animals	The study did not involve wild animals.
Field-collected samples	The study did not involve samples collected from field.
Ethics oversight	All animal experiments were approved by and performed in accordance with the Institutional Animal Care and Use Committees of National University of Singapore (R16-1644 and R20-1586).

Note that full information on the approval of the study protocol must also be provided in the manuscript.

Human research participants

Policy information about studies involving human research participants

Population characteristics	All patient samples are deidentified and we do not have the information about the sex and age of the patients.
Recruitment	A total of 17 matched pairs of primary HCC and adjacent non-tumor (NT) tissues were randomly chosen from the same cohort obtained from the Sun Yat-Sen University Cancer Centre (Guangzhou, China). A total of 20 matched pairs of primary CRC and adjacent NT colon tissues were randomly chosen from the same cohort obtained from the National University Hospital, Singapore.
Ethics oversight	All human tissue samples used in this study were approved by the committees for ethics review at Sun Yat-Sen University, National University of Singapore and the National University Hospital, Singapore.

Note that full information on the approval of the study protocol must also be provided in the manuscript.
